# Prevalence and Screening Rates of Hepatitis B and Hepatitis C Infections in Adult Patients with Solitary Organ Tumors

**DOI:** 10.3390/tropicalmed10090258

**Published:** 2025-09-10

**Authors:** Seyhmus Abakay, Hüseyin Döngelli, Nilay Danış, Halil İbrahim Ellez, Göksel Bengi, Tuğba Yavuzşen, Hüseyin Salih Semiz

**Affiliations:** 1 Department of Internal Medicine, Dokuz Eylul University Hospital, Izmir 35000, Turkey; drabakayseyhmus@gmail.com; 2 Department of Gastroenterology, Dokuz Eylul University Hospital, Izmir 35000, Turkey; nilaydanis17@gmail.com (N.D.); goksel.bengi@deu.edu.tr (G.B.); 3 Department of Medical Oncology, Dokuz Eylul University Hospital, Izmir 35000, Turkey

**Keywords:** chemotherapy, hepatitis B, hepatitis C, neoplasm, prophylaxis, virus reactivation

## Abstract

Background and Aims: Hepatitis B virus (HBV), hepatitis C virus (HCV), and human immunodeficiency virus (HIV) remain significant global public health issues despite advances in their diagnosis and treatment. Our country is in a medium endemic region for HBV. Reactivation can occur during or after immunosuppressive therapy. Therefore, screening patients before treatment is crucial to prevent reactivation. However, pretreatment screening is often insufficiently emphasized in studies. This study aimed to assess the incidence of HBV and pretreatment screening rates in patients with solid organ tumors at our center. Methods: We included patients aged over 18 years who were treated for solid organ tumors at our center between January 2016 and January 2022. Data on age, sex, histopathological diagnosis, and serological parameters were retrospectively collected. Appropriate HBV screening was defined as the assessment of HBsAg, anti-HBs, and anti-HBc IgG levels prior to the initiation of immunosuppressive therapy. Results: In our study, HBsAg testing was requested for 13.3% of the patients, and anti-HCV testing was requested for 13.3%. Among the patients screened for HBV and HCV, the prevalence rates of HBV and HCV infection were 3.3% and 1%, respectively. Conclusions: Our findings reveal inadequate screening rates for HBV and HCV among patients receiving immunosuppressive therapy. Increasing awareness about screening and implementing regular educational programs are crucial to protect patients from reactivation.

## 1. Introduction

Hepatitis B virus (HBV) infection is the most common chronic viral infection in the world [1]. In Turkey, HBsAg positivity has been reported to be 4% [2]. Although studies on this topic in solid organ tumor patients are limited, the prevalence is estimated to be similar to that in the general population [3].

Hepatitis C virus (HCV) is an enveloped ribonucleic acid (RNA) virus. Globally, approximately 70 million people have chronic HCV infection, and 1.7 million new infections occur each year [4].

Human immunodeficiency virus (HIV) is an RNA-structured, enveloped virus [5]. For patients with solid organ malignancies, there is no consensus whether routine screening should be performed according to the guidelines of infectious diseases and oncology associations [6].

Patients with HBV infection may receive immunosuppressive treatments such as chemotherapy, which pose a risk for HBV reactivation. Although HBVr can be asymptomatic in some cases, the risk of acute hepatitis and even death should always be considered. In addition, it can lead to interruption or even termination of cancer treatment [7].

Serologic status is the most important marker for determining and preventing the risk of HBV reactivation [8]. Serologic tests for HBsAg, anti-HBc IgG, and anti-HBs can predict reactivation risk before starting immunosuppressive therapy [9]. However, there is no clear international consensus on this issue. Despite differing opinions, all relevant associations agree that every patient starting immunosuppressive therapy should be screened for HBV infection [10,11,12,13].

Owing to the risk of HBV reactivation in HBsAg-positive patients planned for systemic chemotherapy, antiviral prophylaxis is recommended by current guidelines according to risk stratification. In HBsAg-positive patients, the risk of HBV reactivation is approximately 4–68%, and this rate may vary depending on the chemotherapy regimen and the status of hepatitis serology. Studies have found that HBV reactivation is lower in patients who received antiviral prophylaxis (0.9–31.4%) [14].

In centers treating oncology patients with immunosuppressive therapy, HBV and HCV screening should be performed before treatment, as recommended by guideline. This screening helps detect infections and assess reactivation risk. A literature review revealed varying rates of serology screening across centers. This study aimed to determine the frequency of HBV, HCV, and HIV screening, as well as the prevalence, while raising awareness to prevent HBV reactivation.

## 2. Materials and Methods

### 2.1. Patient Selection and Data Curation

A total of 15,902 patients who were followed up in our center for solid organ tumors between January 2016 and January 2022 were included in the study. The patients’ age, sex, date of diagnosis, histopathological diagnosis, cytotoxic treatment, antiviral prophylaxis status, and values for HBsAg, anti-HBc, anti-HBs, anti-HCV, and HIV ag/ab were retrospectively reviewed. HBsAg, anti-HBc IgG and anti-HBs together for HBV, anti-HCV for HCV and HIV ag/ab for HIV were considered appropriate screening methods. All serological tests were conducted in the International Organization for Standardization (ISO) 15189-accredited central laboratory of our institution [15], which adheres to routine internal quality control standards and regular external validation procedures. Serological detection of HBV, HCV, and HIV was performed by a Cobas e 411 analyzer (Roche Diagnostics GmbH, Mannheim, Germany) operating with the electrochemiluminescence method, using HBsAg II, Anti-HBc, Elecsys Anti-HBs II, Anti-HCV II, and HIV combi PT kits (all Roche Diagnostics GmbH, Mannheim, Germany).

### 2.2. HBV Serologic Classification

Patients were classified according to their hepatitis B virus (HBV) serologic profiles based on the combination of HBsAg, total anti-HBc, and anti-HBs results. Specifically, patients were considered susceptible if all three markers were negative, immunized via vaccine if HBsAg and anti-HBc were negative but anti-HBs was positive, immunized after past HBV infection if HBsAg was negative, anti-HBc was positive, and anti-HBs was positive, chronic inactive infection if HBsAg was positive, isolated anti-HBc IgG positive if only anti-HBc was positive while HBsAg and anti-HBs were negative, and incomplete serologic data/HBV status undetermined if one or more of the three tests were missing.

### 2.3. Risk Stratification for HBV Reactivation

Among the patients whose HBV serological profiles could be determined, risk classification for HBV reactivation was performed on the basis of the serological profile and the chemotherapeutic agent used, following the 2021 Asian Pacific Association for the Study of Liver (APASL) guidelines. The APASL 2021 classification was preferred because it provides a detailed and up-to-date risk stratification, and since our country, similar to the Asia-Pacific region, has an intermediate-to-high prevalence of HBV, we considered this guideline to be the most appropriate for our patient population [12]. Patients were categorized into low-risk (<1%), moderate-risk (1–10%), and high-risk (>10%) groups. Prophylactic implementation data were subsequently analyzed according to the risk group classification.

### 2.4. Ethics

Our study was approved by our institution’s noninterventional Clinical Research Ethics Committee with a decision dated 19 January 2022 and numbered 2022/03-09. This study was conducted in accordance with the Declaration of Helsinki (2000 revision). Given the retrospective nature of the study, the ethics committee waived the requirement for obtaining informed consent.

### 2.5. Statistical Analysis

The analyses in our study were performed using the IBM SPSS Statistics 24 (Statistical Package for Social Sciences version 24) program. Continuous variables with a normal distribution are presented as the mean and standard deviation, and variables with a nonnormal distribution are presented as the median (interquartile range). Categorical parameters are presented as numbers (percentages). Patients with incomplete serological profiles were included in overall prevalence analyses when possible, but were excluded from subgroup analyses requiring full serological data.

## 3. Results

**Figure 1:**
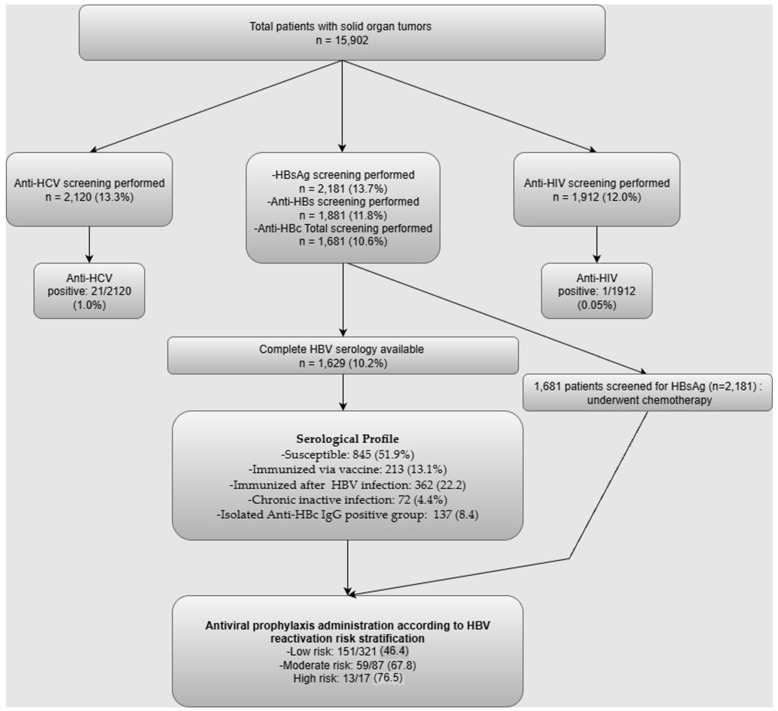
Flowchart of the study.

Among the cohort, HBsAg screening was performed in 2181 patients (13.7% screening prevalence) (Figure 1). Of those screened, 72 patients (3.3% infection prevalence among screened) tested positive for HBsAg, while 2109 (96.7%) were negative. The remaining 13,721 patients (86.3%) were not screened (Table 1).

For anti-HBs, screening was conducted in 1881 patients (11.8% screening prevalence), with 668 (35.5%) testing positive and 1213 (64.5%) negative. For anti-HBc Total, 1681 patients were screened (10.6%), of whom 580 (34.5%) were positive and 1101 (65.5%) negative. In both cases, the majority of patients were not screened (88.2% for anti-HBs, 89.5% for anti-HBc) (Table 1).

Anti-HCV screening was performed in 2120 patients (13.3% screening prevalence), identifying 21 positive cases (1.0% infection prevalence among screened) and 2099 negative. Anti-HIV testing was performed in 1912 patients (12.0%), with only one positive result (0.05% infection prevalence among screened).

The most common primary cancer diagnoses were breast cancer (20.9%), colorectal cancer (17.6%), and lung cancer (16.6%). Other notable cancer types included stomach (6.6%), pancreas (4.0%), bladder (4.9%), and tuboovarian cancers (5.3%). Rare diagnoses included cancers of the thyroid (0.5%), other endocrine glands (0.4%), and testis (1.5%) (Table 1).

Among patients with complete HBV serology, 845 (38.7%) were susceptible, 213 (9.8%) were immunized via vaccination, 362 (16.6%) had immunity following past HBV infection, 72 (3.3%) had chronic inactive infection, and 137 (6.3%) had isolated anti-HBc IgG positivity. In 552 patients (25.3%), only HBsAg was tested, and their HBV immune status remained undetermined (Table 2).

Chronic inactive HBV infection was identified in 3.3% (n = 72) of patients, and 6.3% (n = 137) had isolated anti-HBc IgG positivity. Importantly, in 25.3% of the cohort (n = 552), only HBsAg screening was performed without complete serologic profiling, rendering their HBV immune status undetermined (Table 2).

Among the 2181 patients screened for HBV, a total of 1681 patients who underwent chemotherapy and had complete risk assessment data were classified according to 2021 APASL guidelines HBV prophylaxis risk levels: 323 patients (19.2%) were categorized as low-risk, 87 (5.2%) as moderate-risk, 17 (1.0%) as high-risk, and 1254 (74.6%) as no risk.

The administration of antiviral prophylaxis was evaluated according to HBV reactivation risk categories. Prophylaxis was provided to 13 of 17 patients (76.5%) in the high-risk group, whereas 4 patients (23.5%) did not receive it. In the moderate-risk group, 59 of 87 patients (67.8%) received prophylaxis, while 28 (32.2%) did not. Among low-risk patients, 151 of 323 individuals (46.4%) were administered prophylaxis. Considering the combined moderate- and high-risk cohort of 104 patients, for whom prophylaxis is recommended by various guidelines, 72 patients (69.2%) received antiviral prophylaxis, whereas 32 (30.8%) did not. Detailed risk classification and prophylaxis data are summarized in Table 3.

Owing to the occasional shortages of kits in our center, HBV-DNA monitoring could not be performed, and asymptomatic HBV reactivation could not be detected as a result. In patients investigated for elevated ALT levels, no evidence of reactivation was observed.

## 4. Discussion

In this study, we aimed primarily to determine the screening and infection prevalence of HBV and HCV prior to chemotherapy in patients with solid organ tumors followed in our center. We aimed to assess their risk profiles for HBV reactivation on the basis of the 2021 APASL guidelines and to identify the appropriate and inappropriate practices in antiviral prophylaxis administration according to these risk statuses.

In our study, which evaluated patients with solid organ tumors, HBsAg was positive in 72 (3.3%) of the 2181 patients who underwent HBsAg testing. Of the 2120 patients who were screened for anti-HCV, 21 (1%) were found to be anti-HCV positive. In a similar multicenter study conducted by Koçoğlu et al. in 2018 evaluated 3890 patients with hepatitis B and C serology who were followed up for solid organ tumors [16]. They reported that HBsAg positivity was 3.6%, and anti-HCV positivity was 1.2%, values similar to ours [16]. The seroprevalence rates for HBsAg and anti-HCV antibodies found in our center are consistent with those reported in the literature

The best way to prevent HBV reactivation in patients with solid organ tumors who are treated with immunosuppressive therapy is to screen patients for HBV serology prior to treatment and initiate antiviral prophylaxis in patients with indications. The screening recommendations in the guidelines differ. While the American Association for the Study of Liver Diseases (AASLD) guidelines recommend only HBsAg and anti-HBc IgG for screening purposes, the APASL, American Gastroenterological Association (AGA), European Association for the Study of the Liver (EASL), American Society of Clinical Oncology (ASCO) and Centers for Disease Control and Prevention (CDC) guidelines recommend also testing anti-HBs to identify the group that should be vaccinated early [9,10,11,12,13,17,18].

In patients with solid organ tumors, cytotoxic chemotherapy has been used for many years, but antibody-based and targeted therapies are also being developed, and the frequency of the use of these drugs is increasing. Although HBV reactivation can occur when immunosuppressive therapies are used in people with previous exposure to HBV, screening rates remain low. Surveys conducted in different countries and centers have shown that physicians do not have sufficient awareness of HBV screening. For example, in a survey study conducted by Tran et al. in the United States (US), 265 physicians, including hematologists and oncologists, answered survey questions, and only 20% of the physicians screened for HBV before treatment [19]. In a study conducted by Hwang et al. at MD Anderson Cancer Center in the US, 15,031 patients with solid organ tumors were examined, and only 581 (4%) of the patients were screened for HBV [20].

In our study evaluating patients with solid organ tumors, HBsAg was detected in 2181 (13.3%) of 15,902 patients followed up in our center. Screening rates were 11.8% for anti-HBs and 10.5% for anti-HBc IgG. When the combination of HBsAg, anti-HBs and anti-HBc parameters is defined as appropriate screening for HBV, the number of patients with all three tests performed was 1629 (10.2%).

To prevent HBV reactivation, screening for HBV and determining the risk class on the basis of the serological profile and treatment regimen before chemotherapy are essential. Antiviral prophylaxis is recommended for high-risk patients, individual evaluation is recommended for moderate-risk patients, and monitoring without prophylaxis is recommended for low-risk patients [10,11,12].

Among patients receiving immunosuppressive therapy and screened for HBV, 321 were classified as low risk, 87 as moderate risk, and 17 as high risk for HBV reactivation. In the high-risk group, prophylaxis was initiated in 13 patients (76.5%) and not in 4 patients (23.5%). In the moderate-risk group, 59 patients (67.8%) received prophylaxis, whereas 28 patients (32.2%) did not. In the low-risk group, prophylaxis was administered to 151 patients (46.4%) but not to 172 patients (53.6%).

In the high-risk group, 9 out of 17 patients (52.9%) were diagnosed with breast cancer, making it the most common diagnosis. Some guidelines indicate that the use of anthracycline derivatives in HBsAg-positive patients presents a high risk for HBV reactivation [12]. Given the widespread use of anthracyclines in breast cancer treatment, the high prevalence of breast cancer in this group is likely due to the frequent use of these drugs.

In our study, some high-risk patients who required antiviral prophylaxis did not receive it, whereas some low-risk patients were given unnecessary prophylaxis. Failure to provide prophylaxis in high-risk patients increases the likelihood of HBV reactivation, which may lead to acute hepatitis, interruptions in cancer treatment, and potentially severe or life-threatening complications. Conversely, unnecessary antiviral therapy in low-risk patients can result in avoidable side effects, drug interactions, and polypharmacy, healthcare costs without offering clinical benefit. These inappropriate practices likely stem from limited knowledge of current guidelines, insufficient awareness of HBV reactivation risks, inadequate training, and overly cautious preventive approaches. Our findings underscore the importance of strict adherence to guideline-based risk stratification and tailored prophylaxis, as well as the potential role of targeted educational programs and automated reminders to optimize antiviral use and patient safety.

These results also highlight a broader gap in clinical practice regarding HBV management in oncology. Beyond screening and prophylaxis, there is insufficient emphasis on HBV epidemiology, its natural history, coinfections (such as HCV and HIV), occult hepatitis B infection (OBI), and their clinical consequences. High OBI prevalence has been reported in certain groups, and OBI can both transmit infection and trigger reactivation episodes, posing an additional barrier to achieving the World Health Organization’s 2030 viral hepatitis elimination goals [21]. Further studies addressing OBI and integrating HBV-related education into oncology practice may therefore contribute significantly to prevention strategies.

Although recent guidelines have begun to provide more specific and individualized recommendations, the findings of our study indicate that there are still notable deficiencies, particularly in the implementation of necessary screening procedures for HBV prevention, as well as in the timely initiation of adequate prophylactic treatment [22].

Considering the HBV screening rates in our study and similar studies in the literature, the results are well below the target value. We believe that insufficient knowledge of updated guidelines, poor physician awareness, and occasional inability to perform relevant tests due to systemic failures contribute to these low screening rates.

The absence of HBV-DNA monitoring may have led to underestimation of asymptomatic reactivation cases; however, all patients were monitored via transaminase levels, and no signs of reactivation were observed. This should be considered when interpreting prevalence and prophylaxis results. In addition, the potential adverse effects of unnecessary antiviral prophylaxis, such as drug toxicity, interactions, and polypharmacy, were not systematically evaluated in our study, and this aspect should be addressed in future research.

Finally, as 2030 approaches, the global HBV elimination targets remain out of reach for many countries under current frameworks. While prevention measures have been most successful, improving screening, diagnosis, and timely treatment initiation is urgently needed. Our findings provide insight into local practice gaps but also echo global challenges, underscoring the necessity of aligning oncology practice with broader HBV elimination efforts.

## 5. Conclusions

Our findings, together with existing literature, indicate that HBV and HCV screening rates in patients with solid organ tumors prior to the initiation of treatment remain substantially below recommended levels. To improve compliance with screening protocols, we recommend regular educational programs for healthcare providers on current HBV and HCV guidelines, and integration of automated reminders into hospital electronic medical record systems to ensure timely testing and appropriate antiviral prophylaxis.

## Figures and Tables

**Table 1:** Descriptive and viral serological characteristics of the study groups.

Variables	Total (n = 15,902)
Age *mean* ± *sd*	63.0 ± 13.8
Sex *n* (*%*) Male Female	8332 (52.4%) 7570 (47.6%)
HBsAg *n* (*%*) Positive Negative Screening not performed	72 (0.5) 2109 (13.3) 13,721 (86.2)
Anti-HBs *n* (*%*) Positive Negative Screening not performed	668 (4.2) 1213 (7.6) 14,021 (88.2)
Anti-HBc Total *n* (*%*) Positive Negative Screening not performed	580 (3.6) 1101 (6.9) 14,221 (89.5)
Anti-HCV *n* (*%*) Positive Negative Screening not performed	21 (0.1) 2099 (13.2) 13,782 (86.7)
Anti-HIV *n* (*%*) Positive Negative Screening not performed	1 (<0.1) 1911 (12.0) 13,990 (87.9)
Primary diagnosis (cancer) *n* (*%*) Lung Breast Colorectal Prostate Pancreas Stomach Esophagus Central nervous system Sarcoma Bladder Tuboovarian Hepatobilier Head-neck Testis Thyroid Other endocrine glands Skin Unknown Multiple primer Other	2640 (16.6) 3324 (20.9) 2799 (17.6) 509 (3.2) 636 (4.0) 1049 (6.6) 318 (2.0) 270 (1.7) 429 (2.7) 779 (4.9) 843 (5.3) 350 (2.2) 413 (2.6) 239 (1.5) 79 (0.5) 64 (0.4) 588 (3.7) 48 (0.3) 428 (2.7) 97 (0.7)

**Abbreviations:** anti-HBc, anti-hepatitis B core total antibodies; anti-HBs, antibody to hepatitis B surface antigen; HBsAg, hepatitis B surface antigen; HCV, hepatitis c virus; HIV, human immunodeficiency virus.

**Table 2:** Classification of HBsAg-screened patients by HBV serologic status (n = 2181).

Serological Profile	N	%
Susceptible	845	38.7
Immunized via vaccine	213	9.8
Immunized after HBV infection	362	16.6
Chronic inactive infection	72	3.3
Isolated anti-HBc IgG positive group	137	6.3
Incomplete serologic data; HBV status undetermined	552	25.3

**Abbreviations:** HBsAg, hepatitis B surface antigen; HBV, hepatitis B virus.

**Table 3:** Risk classification for HBV prophylaxis in patients screened for HBV and receiving chemotherapy (n = 427).

Risk Classification	Prophylaxis Applied	No Prophylaxis	Total
High risk	13 (76.5%)	4 (23.5%)	17 (100%)
Moderate risk	59 (67.8%)	28 (32.2%)	87 (100%)
Low risk	151 (46.4%)	172 (53.6%)	323 (100%)

**Abbreviations:** HBV, hepatitis B virus.

## Data Availability

All data used in this study are available from the corresponding author upon request: Hüseyin Döngelli; drhuseyindongelli@gmail.com.

## Abbreviations

The following abbreviations are used in this manuscript: AASLD, American Association for the Study of Liver Diseases; AGA, American Gastroenterological Association; APASL, Asian Pacific Association for the Study of Liver; DNA, deoxyribonucleic acid; CDC, Centers for Disease Control and Prevention; EASL, European Association for the Study of the Liver; HBV, hepatitis B virus; HBVr, HBV reactivation; HCV, hepatitis C virus; HIV, human immunodeficiency virus; RNA, ribonucleic acid; SPSS, Statistical Package for Social Sciences; US, United States.
